# Precoce and opposite response of proteasome activity after acute or chronic exposure of *C*. *elegans* to γ-radiation

**DOI:** 10.1038/s41598-018-29033-1

**Published:** 2018-07-27

**Authors:** Cécile Dubois, Catherine Lecomte, Sébastien Pyr dit Ruys, Mira Kuzmic, Claire Della-Vedova, Nicolas Dubourg, Simon Galas, Sandrine Frelon

**Affiliations:** 1 IRSN/PSE-ENV/SRTE - Laboratoire d’ecotoxicologie des radionucléides - BP3, 13115 St Paul lez Durance Cedex, France; 2 IRSN/PSE-ENV/SRTE – LRTA - BP3, 13115 St Paul lez Durance Cedex, France; 3 IBMM, University of Montpellier, CNRS, ENSCM, Montpellier, France

## Abstract

Species are chronically exposed to ionizing radiation, a natural phenomenon which can be enhanced by human activities. The induced toxicity mechanisms still remain unclear and seem depending on the mode of exposure, *i*.*e*. acute and chronic. To better understand these phenomena, studies need to be conducted both at the subcellular and individual levels. Proteins, functional molecules in organisms, are the targets of oxidative damage (especially *via* their carbonylation (PC)) and are likely to be relevant biomarkers. After exposure of *Caenorhabditis elegans* to either chronic or acute γ rays we showed that hatching success is impacted after acute but not after chronic irradiation. At the molecular level, the carbonylated protein level in relation with dose was slightly different between acute and chronic exposure whereas the proteolytic activity is drastically modified. Indeed, whereas the 20S proteasome activity is inhibited by acute irradiation from 0.5 Gy, it is activated after chronic irradiation from 1 Gy. As expected, the 20S proteasome activity is mainly modified by irradiation whereas the 26S and 30S activity are less changed. This study provides preliminaries clues to understand the role of protein oxidation and proteolytic activity in the radiation-induced molecular mechanisms after chronic *versus* acute irradiation in *C*. *elegans*.

## Introduction

To reach the objectives of environmental radiation protection, it now seems necessary to use integrated approaches to improve the understanding of the mechanisms involved at different levels of biological organization, to find sensitive markers of exposure and ultimately, adequately protect the environment^1–5^. To date, most studies have been focused on the effects of acute ionizing radiation on physiological endpoints and DNA damage^6–8^. However chronic exposure is more relevant to assess environmental risk and cannot be extrapolated from acute exposure. In addition, while DNA damage has been studied for decades, proteins have been much less studied after gamma irradiation. However, proteins are involved in key biological processes, including DNA repair, epigenetic mechanisms, apoptosis and, like DNA, lipids and sugars, are also impacted by ionizing radiation. In particular, their oxidation can become critical for the cell. In addition, proteome protection pathways, particularly those acting against oxidation, can be linked to the differential radiosensitivity of species^9^. One of the most studied oxidative damage to proteins is carbonylation^10^. Protein carbonylation is an irreversible post-translational modification, and is also an aging and oxidative stress biomarker^11^. Protein carbonylation results from the addition of a carbonyl group on the target protein^12^. Protein carbonylation can be induced via four mechanisms of action, such as i) the metal-catalyzed oxidation (MCO) resulting from the generation of highly reactive hydroxyl radicals produced by the Fenton reaction; ii) tryptophan oxidation by oxygen reactive species; iii) the addition of carbonyl groups on the proteins may also be due to the reagents resulting from lipid peroxidation, for example MDA and 4-HNE; and iv) glycoxidation^13^. In addition, it has been shown that after very high doses of gamma rays, an increase of protein carbonylation is correlated i) to the death of *E*. *coli*^14^ and ii) to a decrease of hatching success of *C*. *elegans*^15^. However, to date, there is only data concerning protein carbonylation after huge doses of acute irradiation (up to 200 Gy), and lower doses domain is poorly explored.

Proteins are continuously degraded by proteolytic systems in order to maintain physiological cellular process. Fast removal of proteins is particularly important for damaged proteins. The two main intracellular proteolytic pathways are the ubiquitin-proteasome system (UPS) and autophagy. At the heart of the proteasomal system is the 20S proteasome, with multiple catalytic activities: a trypsin-like activity, a caspase-like activity and a chymotrypsin-like activity that hydrolyze peptide bonds on the carboxyl side of basic, acidic and hydrophobic amino acids^16^.

The 20S proteasome is considered to be physiologically latent, *i*.*e*. unable to degrade normal, tightly folded proteins, because its entrance pores are normally closed. Its activity is modulated by multiple regulators that favor proteolysis by recruiting specific protein substrates, forming the so-called 30S, 26S and 20S proteasomes, and by opening upon binding the entrance pores, thereby allowing substrates to access to the proteolytic active site and to be degraded^17,18^. The 26S/30S proteasomes consist of the 20S core complex bound to either one or two 19S regulatory complex(es) and degrade mainly polyubiquitylated proteins in an ATP-dependent manner^17^.

Despite its latent character, the 20S proteasome can degrade oxidized proteins *in vitro*^19,20^ most likely because they are partially unfolded. Thus it prevents them from aggregating into forms that could become toxic such heavily oxidized and covalently cross-linked aggregates, which are resistant and inhibitory to proteases^20^.

Due to its protein nature, the proteasome itself is a target of ionizing radiation, but studies have only focused on proteasome sensitivity after acute irradiation^21,22^. Regarding sensitivity of proteasome to ionizing radiation, it has been shown that global proteasomal activity is impacted from 2 Gy of acute gamma irradiation in a non-dose-dependent manner^22^.

It has also been established in K562 cells (human chronic myelogenous leukaemia) that under oxidative stress the 26S proteasome tends to degrade the newly oxidized proteins^23^.

This study was then undertaken to initiate a better understanding of the toxicity mechanisms and the response to ionizing radiations. For this approach, the free living nematode *Caenorhabditis elegans*^24^ is a relevant biological invertebrate model in case of chronic exposure because of its short life-cycle and high fecundity, which make it easy to breed under laboratory conditions^25,26^. Finally, *C*. *elegans* is relevant for mechanistic studies, as its genome is fully sequenced^25^ and the molecular pathways partially known. Two groups of *C*. *elegans* were subjected to gamma irradiation, acute *vs* chronic from 0.5 to 200 Gy. The study of the global protein damage (carbonylation) was done in order to assess the sensitivity of this marker after lower doses of gamma radiation that have been tested in the literature; analysis of protein degradation by proteasomes 30S, 26S and 20S was assessed to determine the cellular response level. Finally, in parallel and to make a link between these molecular markers and an ecologically relevant parameter, reproduction of *C*. *elegans* was monitored by measuring the number of progeny per individual and the percentage of hatching.

## Results

### Chronic irradiation

#### Reproduction parameters

Data in Table 1 showed that there is no impact on the percentage of hatching after chronic irradiation from 3 Gy to 6.5 Gy, compared to unexposed worms. On the contrary, the number of progeny per individual was impacted from 3.3 Gy (50 mGy.h^−1^) until 6.5 Gy (100 mGy.h^−1^).

**Table 1:** Number of progeny per individual and percentage of hatching of *C*. *elegans* after chronic irradiation from egg stage to L4-YA stage (65 h). n = 20.

Conditions of irradiation	Control	3 Gy (45 mGy.h^−1^)	3.3 Gy (50 mGy.h^−1^)	4.5 Gy (75 mGy.h^−1^)	6.5 Gy (100 mGy.h^−1^)
Number of progeny per individual	204 ± 58	175 ± 47	142 ± 50*	161 ± 34*	149 ± 49*
% of hatching	100% ± 10	99% ± 1	99% ± 5	98% ± 5	98% ± 1

Data are expressed as means ± SD (*P < 0.05, Dunnet test). Five conditions of irradiation have been detailed i.e. control and irradiated worms. Respective dose (Gy) and dose rate (mGy.h^−1^) are noticed for each one.

These results corroborate with previous studies showing an absence of effect on hatching success on one generation of *C*. *elegans* exposed from 2.5 to 22 Gy^27^ and a decrease of progeny number from 4 Gy (64.5 mGy.h^−1^) at the first generation, worsened with successive irradiated generations *i*.*e*. from 2.5 Gy (42 mGy.h^−1^) at the third generation. Other studies have shown reprotoxicity of chronic gamma irradiation on other organisms than *C*. *elegans*. These studies revealed a decrease of progeny number per individual and a decrease of hatching success on *Ophryotrocha diadema*, *Neanthes arenaceodentata*, *Daphnia magna* and *Eisenia fetida* exposed to 0.19 mGy.h^−1^, 13.7 mGy.h^−1^, 31 mGy.h^−1^ and 43 mGy.h^−1^ respectively^28–30^. These results showed an increase in accumulation of DNA damage in non-dividing cells in the case of chronic exposures. These damage became apparent after fertilization when the cells started to divide, leading to a decrease of hatching success^28^. Nethertheless, these organisms have longer periods of gametogenesis and different modes of reproduction than *C*. *elegans*. In the present study, the effect on the number of progeny per individual from 50 mGy.h^−1^, could be explained by a gametogenesis failure as growth is not impacted^27^. To better understand, the contribution of molecular mechanisms in the reproduction decline after gamma irradiation on *C*. *elegans*, protein damage and its turnover, were then assessed.

### Protein carbonylation production and degradation by proteasome

**Figure 1:**
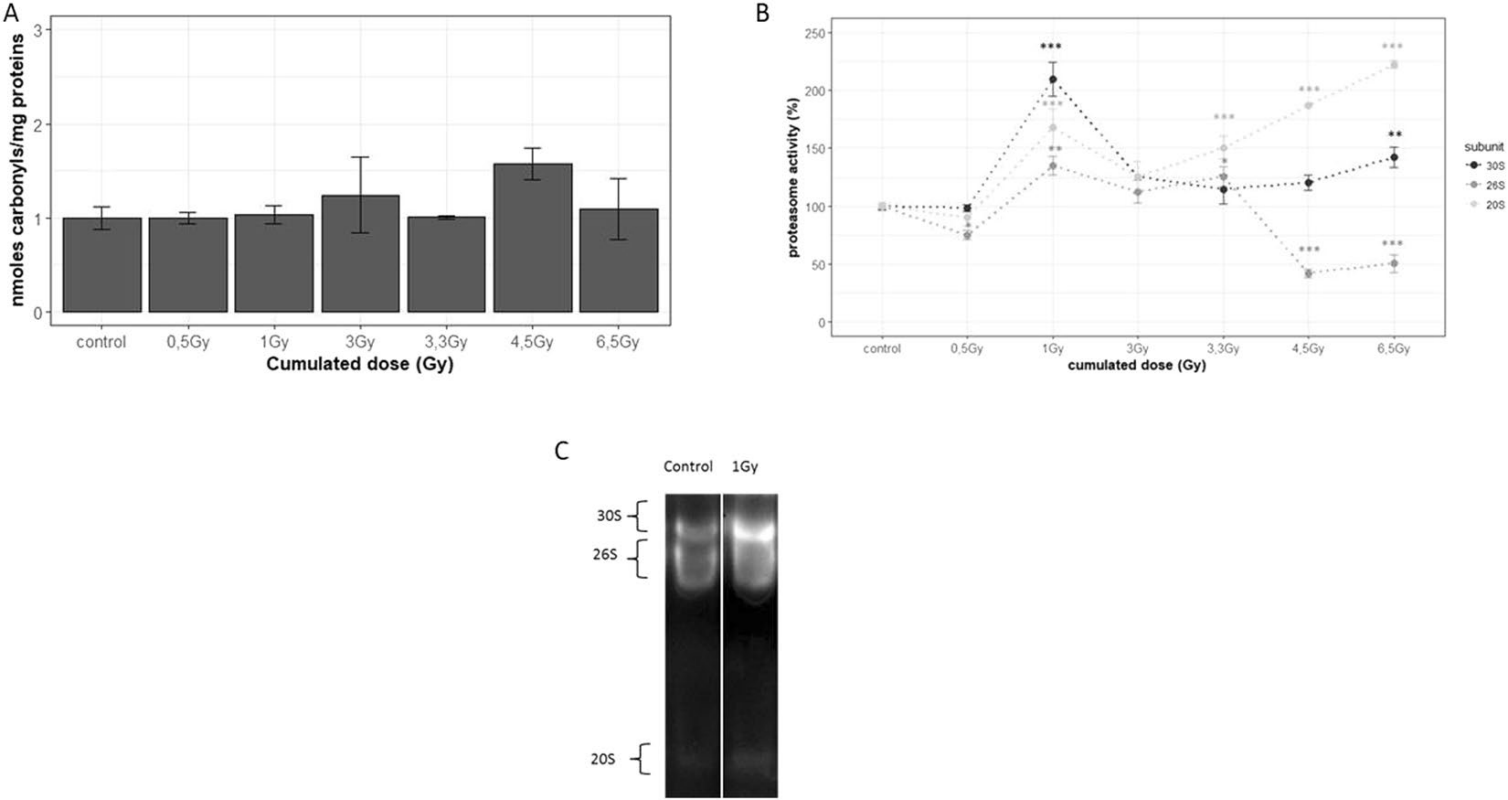
A/Dose response curve of protein carbonylation induction after chronic irradiation from 0.5 to 6.5 Gy. Worms used in this assay were 3 hours synchronized, irradiated from 0.5 to 6.5 Gy (cumulated doses), and collected. n = 3 (pool of 1000 nematodes). Data are expressed as means of protein carbonylation level (induction relative to unexposed worms) ± SEM (*P < 0.05 Dunnet test). B/Dose response curve of proteasome activity after chronic irradiation from 0.5 to 6.5 Gy. Native gel electrophoresis followed by quantification using IQTL software. n = 3 (pool of 3000 nematodes). Data are expressed as means of proteasome activity (% relative to unexposed worms) ± SEM for the 30S (dark grey), 26S (middle grey) and 20S (light grey) proteasomes as (*p < 0.05; **p < 0.01; ***p < 0.001, Dunnet Test). B/Native gel electrophoresis, followed by in gel proteasome activity assay of the 30S 26S and 20S proteasomes; white line delimits the cropped zone from the same gel (full-length gel is presented in supplementary file Figure S1).

Protein carbonylation level was monitored after chronic irradiation from 0.5 to 6.5 Gy (7 to 100 mGy.h^−1^). No significant difference was observed between conditions (Fig. 1A). Proteasome activity and its dissociation upon chronic exposure to gamma rays were also measured. Interestingly, as presented in Fig. 1B, in gel activity of each subunit (measured as shown in Fig. 1C, full-length gel is presented in supplementary file Figure S1) allowed to detect a significant increase of the 30S proteasome activity at 1 Gy (+110%) and at 6.5 Gy (+37%) (respective *p*.*values* = 1.2.10^−7^, 0.008), and of the 26S proteasome activity at 1 Gy (+34%) and 3.3 Gy (+22%) (respective *p*.*values* = 0.001, 0.1) compared to controls. On another hand a significant decrease of the 26S proteasome activity was found at 0.5 Gy (−25%), 4.5 Gy (−56%) and 6.5 Gy (−48%) (with respective *p*. *values* = 0.01, 3.10^−6^, 2.10^−5^). Interestingly, the activity of the 20S proteasome increased between 20 and 55% compared to unexposed worms from 1 Gy (respective *p*.*values* from 1 to 6.5 Gy of 1.10^−5^, 5.10^−4^, 0.05, 2.10^−7^, 5.10^−9^).

The carbonylated protein level measured in our study depends on the balance between its production and its degradation. The fact that the level of carbonylated protein after chronic exposure from 0.5 to 6.5 Gy is quite stable can be due to 3 phenomenon; it is possible that chronic irradiation i) does not induce an increased production of carbonylated proteins, ii) induces an activation of antioxidant molecules, or iii) induces degradation of carbonylated proteins. Through our results showing that the 30S, 26S and 20S proteasome activities are increased at 1 Gy with no modulation of the carbonylated protein level, it can be supposed that from 1 Gy the proteasome burst observed is sufficient to remove the carbonylated proteins produced, leading to a stable balance between carbonylated protein production and degradation.

Results showed that chronic irradiation leads to a deep remodeling of the equilibrium between the different forms of the proteasome, especially at the highest cumulated doses. Equivalent to 38%, 55% and 6% of activity for 30S, 26S and 20S at the basal level, respectively, the proteasomal activity distribution after the highest dose of γ-rays, *i*.*e*. 6.5 Gy, is 58%, 29% and 13% of activity for 30S, 26S and 20S. This suggests a compensation of these three proteasome forms in order to maintain a basal level of global proteasome activity. After chronic irradiation, proteolysis seemed to be stimulated in order to remove oxidized proteins. This phenomenon has already been observed in condition of mild oxidative stress where the 20S proteasome was responsible for the degradation of oxidatively damaged histones to promote the turnover of these proteins, indirectly allowing the efficient reparation of DNA damage^31^. After gamma radiation, the process could be the same as reactive oxygen species that can oxidize histones are also produced. Indeed, the activation of the 20S proteasome activity could be due to the recruitment of proteasome activators (PA28α), as it has been previously shown during mouse embryonic stem cells differentiation^32^. This phenomenon could be relevant in our study, as nematodes were exposed to gamma radiation from egg stage to L4-YA stage, covering the differentiation of embryonic stem cells. Moreover, the increase of the 20S activity could be also due to the dissociation of the 30S and 26S proteasomes in order to increase the level of the free 20S proteasome^33^. This phenomenon seems to be preponderant in our study as 26S and 20S proteasome activities seemed distinct, and especially 26S proteasome activity decreased whereas 20S activity increased from 4.5 Gy. As described in a precedent study, stress-induced disassembly of the 30S and 26S proteasomes is used to increase the 20S expression level, allowing cells to clear more effectively the damaged proteins and mitigate the cytotoxic effects of their accumulation. In fact, a recent study has shown that cysteine (amino acid target of carbonylation^13^) oxidation leads to proteasome dissociation under oxidative stress^34^. In this manner, cysteine modifications may influence the ratio of 20S to 26S activities. However, as 30S proteasome activity seemed to have different behavior than the 26S proteasome, we posit that as the 30S has a more important activity than the 26S, it is possible that a decrease of the 26S activity serves to recruit free 19S (by dissociation) to form new 30S proteasomes in order to promote a rapid turnover of DNA reparation enzymes by the 30S proteasome.

Both ubiquitin-dependent proteasomes 26S et 30S are known to be involved in the DNA damage responses pathways (the so-called “DDR pathway”), by allowing the degradation and the renewal of proteins necessary to DNA damage reparation^35,36^. The stimulation of their activities from 1 Gy, and particularly of the 30S proteasome could possibly belong to this DNA damage repair pathway after a continuous stress such chronic irradiation. Moreover, our results are consistent with literature which has shown that proteasome activation is involved in a decrease of carbonylated proteins and leading to an increased resistance to oxidative stress^37^.

### Acute irradiation

#### Reproduction parameters

A significant decrease of the number of progeny per individual, compared to control, was observed from and above 50 Gy, associated with a decreased rate of hatchability in a dose-dependant manner, until 200 Gy (Table 2). The ED50_number of progeny per individual_ and the ED50_rate of hatching_ were calculated *via* a log-logistic modelling curve to be 79.8 Gy (CI95 = [47.4; 112.1]) and 77.4 Gy (IC95 = [70.4; 84.4]), respectively.

**Table 2:** Number of progeny per individual and percentage of hatching of *C*. *elegans* after acute irradiation at the L4-YA stage. n = 20.

Conditions of irradiation	Control	2.5 Gy	6.5 Gy	14.4 Gy	50 Gy	100 Gy	200 Gy
Number of progeny per individual	173 ± 15	190 ± 54	143 ± 47	168 ± 29	113 ± 28*	79 ± 46**	22 ± 5***
% of hatching	98% ± 2	96% ± 3	96% ± 5	95% ± 4	81% ± 7*	26% ± 19**	0% ± 0***

Data are expressed as means ± SD (*P < 0.05; ** < 0.01; ***P < 0.001, Dunnet Test).

Our results were consistent with previous studies on *C*. *elegans* which showed that the number of progeny per individual is impacted from 30 Gy and a decrease of hatching success is observed from 40 Gy of acute irradiation^6,38^. These findings suggest that unlike chronic irradiation, both gametogenesis and embryogenesis may be impacted after acute irradiation as previously shown^38^. Other studies carried out using cesium-137 source on invertebrates, showed that the hatching success of *Neanthes arenaceodentata* is impacted from 0.5 Gy, and that the DE50_rate of hatching_ (Dose inducing 50% of Effect compared to controls) for *Folsomia candida* and *Eisenia fetida* was 21.9 Gy and 11.1 Gy respectively^28,39,40^. This suggests that *C*. *elegans* is less sensitive to ionizing radiation than these other invertebrates. These studies suggest that reduction in hatching success may result from lethal mutations in the developing gametes, which affects the survival of embryos at early life stage^28^. The same phenomenon can be suspected for *C*. *elegans*, with an induction of deleterious mutations during gametogenesis by acute irradiation, which are not repaired and are then transmitted to developing embryos^38^. About the number of progeny decrease, one explanation can be an increase of gamete apoptosis due to radiation damage^41^. Similarly to chronic study, investigations at the protein level and proteolysis have been done to better understand the phenomenon.

#### Set up of the experimental design for carbonylated protein measurement after acute exposure

Basal level of carbonylated proteins in *C. elegans* over its life cycle: Several studies highlighted that increased protein carbonyl content is a hallmark of cellular and organism aging^42–44^. As *C*. *elegans* has a very rapid life cycle and changes its life stage within a few hours, also involving important metabolic variation during these stage switches (gonad establishment, increased protein biosynthesis and mitochondrial level), the analysis of the basal level of carbonylated proteins in the nematode over a part of its life cycle was necessary in order to define the most relevant life stage to measure carbonylated protein level. Indeed, protein carbonylation level was measured at different life stages, in order to identify the stage with the lowest protein carbonylation (PC) level to be in the best signal to noise ratio situation and therefore improve the sensitivity of this PC marker for future irradiation experiments.

**Figure 2:**
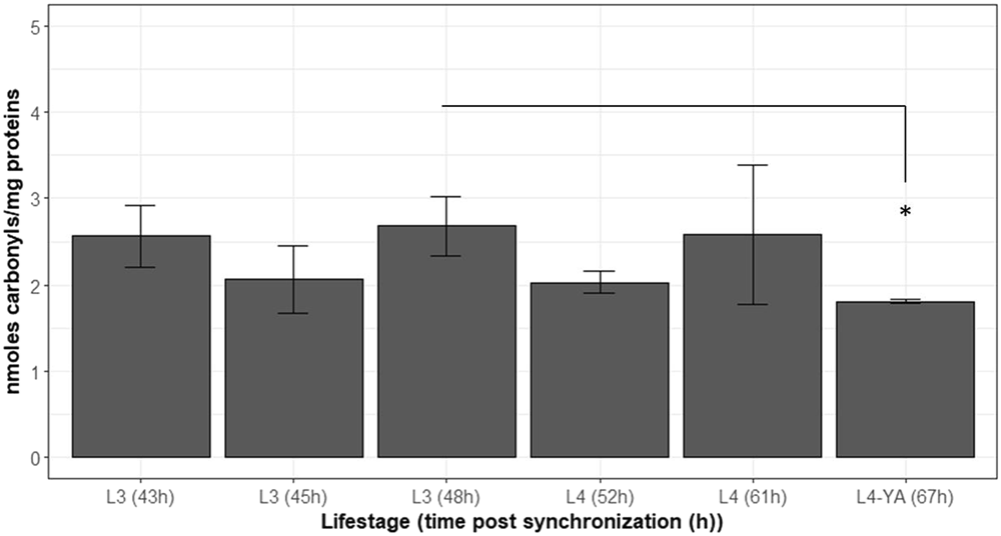
Basal level of carbonylated proteins over *C*. *elegans* lifecycle (L3, L4 and L4/YA stages). Times in brackets correspond to time post synchronization (in hours). Data are expressed as means of carbonylated protein level (nmoles carbonyls per mg of proteins) ± SD as (*p < 0.05, Tukey Test).

Testing the different life stages (L3, L4 and L4-YA stages) showed that the carbonylated protein level is around 1.9 ng of carbonyls/mg of proteins (±0.09) and varied moderately during *C*. *elegans* life cycle (Fig. 2). The only significant difference was between the L3 (48 h post synchronization) and the L4-YA (67 h post synchronization) stages (*p*. *value* = 0.03), showing that the L3 stage has the highest basal level of carbonylated proteins. In conclusion, results showed a low but gradual decrease of the basal level of carbonylated proteins from the L3 to the L4-YA adult stages.

Several processes may explain the observed difference between the L3 and the L4-YA stage. First of all, the L3 stage is marked by the initiation of the prophase I of meiosis, the gonad arms undergone a rapid extension, meaning an increased need for cellular energy^45^. The L3 stage is the life stage where mitochondria are the most numerous. Yet, mitochondria are also a stock of reactive oxygen species that could thus oxidized proteins and produce carbonylated proteins, even if the Ion protease of the mitochondria is involved in the removal of damaged proteins^12^,^46–48^. Conversely, at the end of the L4-YA stage, somatic cellular division stopped and gonads are well established. The low level of carbonylated proteins at the L4-YA stage can therefore be linked to this lower production of reactive oxygen species but also to an increased proteolytic activity in order to remove carbonylated proteins. Further studies will be necessary to investigate the role of the proteasome in the carbonylated protein level variation during *C*. *elegans* lifecycle. However, the L4-YA stage was used in this study because of its low biological variability in terms of protein carbonylation and because of previous experiments run at this stage in the laboratory for chronic irradiation^27^.

Kinetics of carbonylated protein production after acute irradiation: Kinetics of carbonylated protein production after acute irradiation is very little documented and the optimum time at which the analysis must be made is poorly described. Kinetics of carbonylated proteins production were therefore measured after two different doses (ED50 acute reproduction and a lower dose, close to the chronic dose associated to a reproduction effect) and dose rates of acute irradiation to determine an optimum time post-irradiation for the sampling and the analysis of carbonylated protein level, resulting from the balance between their production (proportional to the ROS) and their elimination (in connection with cellular proteolytic activity).

Results in Table 3 showed a significant induction of carbonylated proteins level compared to controls at 3 hours post irradiation at 2.5 Gy (*p*. *value* = 0.03). A decrease of carbonylated protein level seemed to occur at 6 hours post-irradiation but the high standard deviation at 3 hours post irradiation prevented statistical validation of this conclusion. At 75 Gy a significant increase was observed at 2 hours 40, 4 hours and 5 hours post irradiation compared to controls (respective *p*. *values *= 4.10^−5^; 3.10^−5^; 1.10^−5^). The maximum accumulation of carbonylated protein seemed to occur between 2h40 and 5 hours post irradiation. Furthermore, for the two doses, carbonylated protein induction was very similar despite the dose rate difference. The optimal time for protein carbonylation analysis was therefore chosen at 3 hours post irradiation since protein carbonylation level was maximum at this time for the two very different doses and dose rates tested.

**Table 3:** Kinetics of carbonylated proteins production on *C*. *elegans* after acute irradiation at 2.5 Gy (1 Gy.min^−1^) and at 75 Gy (15 Gy.min^−1^).

Time post irradiation (h)	Control	1 h	1h30	2 h	2h40	3 h	4 h	5 h	6 h
2.5 Gy	1 ± 0.1	1.2 ± 0.2	0.9 ± 0.1	1.6 ± 0.4	NA	2.0 ± 0.7*	NA	1.9 ± 0.7	1.3 ± 0.2
75 Gy	1 ± 0.2	1.2 ± 0.2	0.8 ± 0.2	NA	2.0 ± 0.2***	NA	1.9 ± 0.1***	2.1 ± 0.3***	NA

Worms used in this assay were 3 hours synchronized, irradiated at 2.5 and 75 Gy, and collected at different times post irradiation as indicated. n = 3 (pool of 1000 nematodes). Data are expressed as induction (ratio of carbonylated protein level between exposed and controls) of carbonylated proteins compared to controls as means ± SD (*P < 0.05; **P < 0.01; ***P < 0.001, Dunnet test). NA corresponds to No Available Data.

#### Protein carbonylation production and degradation by proteasome after acute exposure

**Figure 3:**
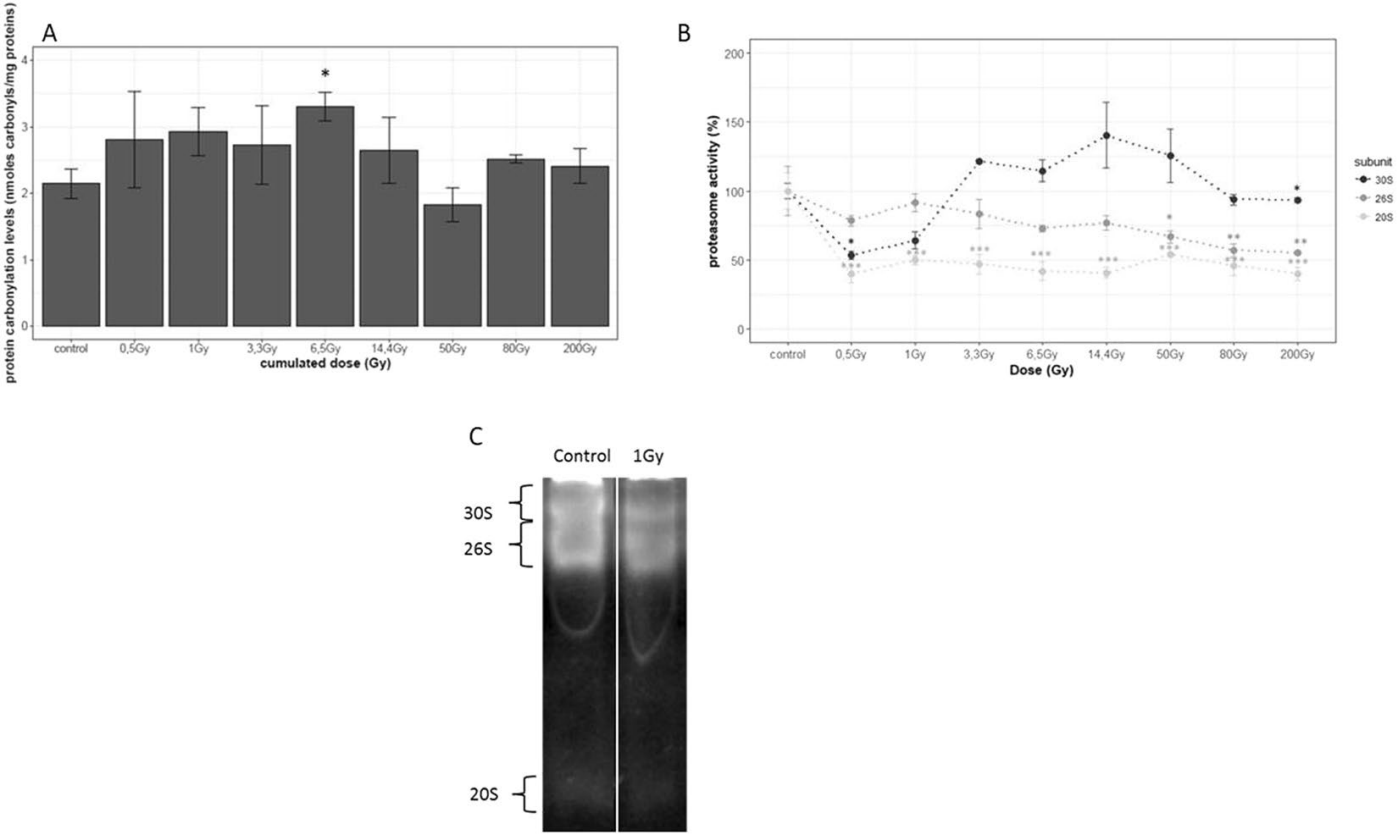
A/Dose response curve of protein carbonylation induction after acute irradiation from 0.5 to 200 Gy. Worms used in this assay were 3 hours synchronized, irradiated from 0.5 to 200 Gy (1 Gy.min^−1^ and 15 Gy.min^−1^), and collected at 3 h post irradiation. n = 3 (pool of 1000 nematodes). Data are expressed as means of protein carbonylation level (induction relative to unexposed worms) ± SEM (*P < 0.05 Dunnet test). B/Dose response curve of proteasome activity from 0.5 to 200 Gy. Native gel electrophoresis followed by quantification using IQTL software. n = 3 (pool of 3000 nematodes). Data are expressed as means of proteasome activity (% relative to unexposed worms) ± SEM for the 30S (dark grey), 26S (middle grey) and 20S (light grey) proteasomes as (*p < 0.05; **p < 0.01; ***p < 0.001, Dunnet Test). C/Native gel electrophoresis followed by in gel proteasome activity assay of the 30S 26S and 20S proteasomes; white line delimits the cropped zone from the same gel (full-length gel is presented in supplementary file Figure S2).

Once the optimum experimental conditions *i*.*e*. life stage to be irradiated, post-irradiation sampling time, were determined, L4-YA nematodes were exposed from 0.5 to 200 Gy (respective dose rates of 1 Gy.min^−1^ from 0.5 to 14.4 Gy doses and 15 Gy.min^−1^, from 50 to 200 Gy doses) of acute irradiation. The results in Fig. 3A only showed a significant increase of protein carbonylation level for the 6.5 Gy dose compared to controls (*p*. *value* = 0.01). These results, as well as the ones for kinetics of carbonylated protein levels, demonstrated that the carbonylated protein level response was not proportional to the dose. In our study, we showed that the induction of carbonylated proteins after acute irradiation is small (maximum of 2.0 ± 0.2 at 75 Gy).

Krisko and collaborators^15^ have previously measured the carbonylated protein level after acute irradiation from 200 Gy to 6000 Gy on *C*. *elegans* (respective dose rates of 26 and 38 Gy.min^−1^). In their study, at 200 Gy, the induction rate compared to controls was 3.3, and then increased to reach a plateau of 4.6 from 900 to 6000 Gy. This drastic increase of the level of carbonylated proteins could be due to a saturation of the cellular defense mechanisms such as the proteolysis systems, degrading oxidized proteins, or the systems against reactive oxygen species. This difference with our results may be due to the dose rate range used by Krisko and collaborator. Previous studies showed that carbonylated proteins are mostly eliminated by proteasome^12^,^19,49^ but this latter is also a target of irradiation and can be impacted from 2 Gy^22^. To test such hypotheses, the activities of the main forms of the proteasome and proteasome dissociation were assessed after native electrophoresis, as done previously after chronic irradiation (Fig. 3C, full-length gel is presented in supplementary file Figure S2). Results in Fig. 3B showed a non-linear dose-response relationship for the 30S proteasome as its activity is significantly decreased at 0.5 (−46%) and 200 Gy (−19%) compared to unexposed worms, but not between 33 and 80 Gy. These results on the 30S proteasome seemed to corroborate with the hypothesis of non-linear effects with threshold, which showed that especially at low doses, a discontinuous dose-response relationship can occur^50^. This is particularly interesting in terms of radiobiology, and even more for environmental risk assessment of ionizing radiation.

The 26S proteasome activity was significantly decreased from 50 Gy to 200 Gy (from −15% to −30%) compared to unexposed worms. Finally, the 20S proteasome activity was significantly lowered from 0.5 Gy in a non-dose-dependent manner until 200 Gy (from −30% to −57%) compared to unexposed worms. At this step, there is no proven link between proteasome activity decline and carbonylated proteins increase at 6.5 Gy compared to controls. Moreover, complementary studies will be needed in order to understand the involvement of proteasome activity in the observed effects on carbonylated proteins level after gamma radiation exposure (by using mutants that exhibit proteasome activity for example).

Finally, the sum of the 3 main proteasome form activities (mostly due to the 30S and the 26S activities) was not so modified in our conditions, *i*.*e*. 3 hours post irradiation, from 0,5 Gy to 14,4 Gy, showing that lower doses than 50 Gy of acute irradiation do not induce dissociation of the proteasome complex. Our results differ from than another study which demonstrated that global proteasome activity of human prostate carcinoma cells was impacted from 2 Gy (5 Gy.min^−1^) at 3 hours post irradiation in a non-dependent relationship^22^. But this difference can be explained by the use of two totally different biological models (*in vivo vs in vitro*; invertebrate *vs* superior vertebrate, and healthy *vs* cancer cells)^51^. In their study however, irradiation was then shown as a proteasome inhibitor then possibly yielding an accumulation of the pro-apoptotic p53 protein (the ortholog of p53 in *C*. *elegans* is CEP-1^52^) and an increase of apoptosis leading to tumor destruction^53^. The link between apoptosis, p53 mediated, and the blocking of proteasome activity has been studied in multiple cell types^54–56^ and notably because p53 is degraded by the ubiquitin proteasome system^57,58^. After acute irradiation exposure of *C*. *elegans*, apoptosis has been shown to increase from 7.5 to 120 Gy^7^, which could be consistent with the decrease in our study of the 26S proteasome activity from 50 Gy. In addition, Bailly and Gartner have shown that in *C*. *elegans* germline this apoptosis was linked to increase of DNA- Double Strand Breaks (DSB)^7^ which could partly explain the reproduction failure found in our study, confirming an impact on both gametogenesis and embryogenesis. The 20S proteasome is known to be less sensitive to UV radiation and low doses of H_2_O_2_ (concentrations below 2 mM were slightly stimulatory) than the 30 and the 26S proteasomes^59–61^, indicating that its proteolytic sites are not targets of mild oxidative stress and UV-damage. But our study shows that it seems sensitive to acute gamma radiations. The 20S active core is known to selectively degrade damaged proteins, included oxidized ones^19,61,62^. In our study, the 20S proteasome activity, as well as the 26S proteasome, seemed to be overtaken, that does not allow them to effectively degrade carbonylated proteins after acute irradiation from 0.5 to 200 Gy. The level of carbonylated proteins being constant despite the inhibition of the 20S activity from 0.5 Gy, of the 26S from 50 Gy, and of the 30S at 200 Gy, another regulation mechanism of carbonylated proteins was probably involved. It has also been shown that the ubiquitin-proteasome system is functionally linked with autophagy, a second major intracellular protein degradation system^63^. Autophagy is usually responsible for the degradation of long-lived proteins and other cellular components, but evidence suggests that this system also plays compensatory role and is activated by ubiquitin-proteasome inhibition^64^. In addition a study highlighted that the proteasomal system degrades proteins one by one whereas autophagy degrades protein aggregates^65^. This provides a possible mechanism likely to degrade carbonylated protein aggregates explaining why the induction rate of carbonylated proteins after acute irradiation was small^66,67^.

## Discussion

In this study we assessed the mechanisms involved in the acute *vs* chronic responses to gamma irradiation by studying protein oxidation, proteasome response and their link to the observed effects on *C*. *elegans* reproduction parameters. We observed that for a same cumulated dose, chronic irradiation may have less impact on embryo development than acute irradiation, and higher impact on the number of progeny per individual (lower cumulative dose). As our results did not show any dose-dependent correlation between carbonylated protein level and reproduction decrease in both irradiation modes, it does not seem that carbonylated protein level can be directly linked neither to total brood size nor to the hatching success decline.

After acute irradiation, our study showed that 26S proteasome activity and reproduction parameters are impacted from 50 Gy. Apoptosis is a highly conserved mechanism used to eliminate superfluous or damaged cells in multicellular eukaryotes; it is remarkably prevalent in germ cells and activated by p53. Indeed, germ cells are dedicated for the maintenance of both eternal proliferative potential and pluripotency to allow the differentiation after fertilization whereas somatic cells are dedicated to contribute to the fitness for one generation. Supervising mechanisms are thus likely to be extremely important in the germline to ensure quality control. It is well known that an increase of apoptosis is linked to reproduction decline^41^, more particularly on the number of progeny. On the contrary, activities of the three forms of proteasome declined at different doses, possibly leading to a defect in the DDR pathway contrary to chronic irradiation, leading therefore to apoptosis possibly explaining the decrease of the hatching success after acute irradiation on *C*. *elegans* from 50 Gy.

## Conclusion

Our results confirm that the effects induced by chronic irradiation differ in quality and intensity from those induced by acute irradiation, thus highlighting the limitations of the extrapolation of data obtained for acute exposure in order to predict the effects of chronic exposure.

In addition, the protein carbonylation level is not a sensitive and predictive marker of the effects of reproduction neither after acute nor after chronic irradiation. However, the 20S proteasome activity was also more sensitive than reproduction parameter for acute and chronic irradiation (impact on the 20S proteasome activity from 0.5 Gy and 1 Gy respectively). The use of this molecular marker seems to be complementary in term of sensitivity and biological relevance to reproduction measurement after irradiation. Moreover, to our knowledge these results show for the first time that chronic irradiation leads to a stimulation of the proteasome activity *in vivo*, which could lead futures directions to the understanding of molecular mechanisms involved after chronic exposure.

The results of the present study provide the preliminary clues to understand the role of protein oxidation and their degradation in the radiation-induced molecular mechanisms after chronic *versus* acute irradiation in *C*. *elegans*.

Further studies will be necessary to determine a molecular damage mapping and to determine the extent to which the ubiquitin proteasome activity could potentiate or enable repair of DNA damage, apoptosis and autophagy after gamma irradiation. This could have an important implication for environmental biomonitoring of chronic exposure to ionizing radiation.

## Material and Methods

### Strain and maintenance conditions

The wild-type N2 strain of *C*. *elegans* provided by CGC (*Caenorhabditis* Genetic Center) was used in this study. Populations were maintained at 19 °C and 80% of humidity on 9 cm petri dishes poured with NGM (Nematode Growth Medium) and seeded with *Escherichia Coli OP50* as food source.

*E*. *coli* OP50 were grown in L-Browth medium at 37 °C overnight. Petri dishes were seeded with 1 mL of saturated culture of bacteria and UV killed (Bio-Link Crosslinker, λ = 254 nm; intensity = 200 mWm^−2^) for 20 minutes to avoid food heterogeneity between dishes.

### Age synchronization of *C*. *elegans*

#### Age synchronization for irradiation

100 gravid worms were randomly selected from the stock population and placed on 9 cm petri dishes. 96 h later, eggs were separated from adult worms by a bleaching procedure^68^ and collected embryos were allowed to grow in a control incubator for 96 h. The gravid worms were separated from eggs already laid by a sucrose gradient (3.5–7%), and then re-synchronized by a bleaching procedure in order to collect the eggs *in utero* synchronized over 3 h.

#### Age synchronization for studying the basal level of carbonylated protein over C. elegans life cycle

In order to measure the basal level of carbonylated proteins on *C*. *elegans*, 1000 synchronized embryos were allowed to growth in a control incubator (19 °C and 80% humidity). Worms were then collected at different times post-synchronization (48 h, 52 h and 67 h) corresponding respectively to the L3, L4 and L4-YA stages and snap frozen.

### Irradiation

Irradiations were performed in control incubators (19 °C and 80% humidity), data loggers were placed in the incubator in order to measure humidity and temperature during irradiation. Nematode plates were placed perpendicularly to the cesium-137 source to obtain an homogeneous dose rate at the surface of the plate. Radio Photo Luminescent dosimeters (RPL, GD-301 type, Chiyoda Technol Cor-poration, Japan) were placed on each experimental unit in order to measure the delivered cumulated dose. At the end of each irradiation, worms were collected, rinsed with M9 medium (5 g.L^−1^ NaCl, 25 mM KPO_4_ buffer and 1 mM MgSO_4_) to ensure bacteria removal, centrifuged and the pellets were snap frozen.

#### Acute

For acute irradiation, 3000 age-synchronized embryos were transferred to fresh 6 cm plates and allowed to reach L4-YA stage in a control incubator. Nematodes were then irradiated with a cesium-137 source (200 TBq) using the GSR-D1 apparatus from RadExpe platform (Curie Institute, France). L4-YA *C*. *elegans* were irradiated at two different dose rates (1 Gy.min^−1^ and 15 Gy.min^−1^ depending on the sample position, to keep the irradiation time equivalent) during different times of irradiation in order to test ten cumulated doses (excluding control): 0.5, 1, 2.5, 3.3, 6.5, 14.4, 50, 75, 80 and 200 Gy.

#### Chronic

For chronic irradiation, nematodes were exposed to cesium-137 source using the platforms MIRE (Mini Irradiator for Radio-Ecology) (1.6 GBq) and MICADO-Lab (Moyen d’Irradiation Chronique pour l’Acquisition de relations Dose-effet en Laboratoire) (370 GBq) (Cadarache, France), from embryo stage to L4-YA adult stage to cover the complete lifecycle (65 h). Six dose rates (excluding controls): 7, 14, 45, 50, 75 and 100 mGy.h^−1^ corresponding to six cumulated doses (0.5, 1, 3, 3.3, 4.5, 6.5 Gy) were tested. Chronic irradiations at 7, 14 and 50 mGy.h^−1^ were performed using MIRE facility, whereas 45, 75 and 100 mGy.h^−1^ were reached by using MICADO-lab facility.

### Reproduction measurements

Reproduction was observed daily until the end of spawning (8 days). To monitor brood size, 20 worms per condition were daily transferred into 20 individual petri dishes. Petri dishes with eggs were placed at 19 °C during 15 h to allow hatchability. The hatched progeny and the unhatched eggs were counted. This method allows measuring number of progeny per individual and hatching success.

### Protein extraction and protein carbonyl content measurement

Kinetics of carbonylated protein production were assessed with data obtained at 2.5 Gy (1 Gy.min^−1^) and at 75 Gy (15 Gy.min^−1^) with worm sampling from 1 hour to 6 hours post-irradiation. Only one control has been carried out (time = 1h30) because all nematodes were within the same life stage and thus the same basal level of carbonylated proteins.

After irradiation, 1000 *C*. *elegans* per replicate were subjected to protein extraction. 100 µl of 0.5-mm diameter zirconium beads and an equal amount of lysis buffer (30 mM Tris–HCl pH 7.4, 150 mM NaCl, 1.0%(v/v) Igepal CA-630 (NP-40), 1%(v/v) TritonX-100, 0.5% (w/v) sodiumdeoxycholate, 0.1%(w/v) sodium dodecyl sulfate (SDS), 2%(v/v) glycerol, 2 mM 1,4-dithiothreitol (DTT), 1 mg.ml^−1^ leupeptin, 1 mg.ml^−1^ aprotinin, 1 mM phenylmethylsulfonylfluoride, 1 mM ethylenediaminetetraaceticacid (EDTA)) were added on top of worm pellets and incubated for 15 min on ice. *C*. *elegans* were then homogenized by three 6800-rpm cycles in the Precellys grinder system (Bertin Technologies, Montigny-le-Bretonneux). After 1 h incubation on ice, lysates were centrifuged (13500 g) at 4 °C for 15 min. Supernatant was sampled, protein concentration was determined using the BCA kit (ThermoScientific) using BSA (Bovine serum albumin) as a standard, according to the manufacturer’s instructions and the remaining volume quickfrozen with liquid nitrogen.

Protein carbonylation levels were determined as described before^69^. Briefly, 15 µg of proteins were labeled by the addition the carbonyl labelling dye (Cy5™-Hz MW569.61 g/mol (FP-IO2490, Interchim) for 20 min at 25 °C in the dark at the final concentration of 0.25 mM. Samples were then precipitated on ice for 20 min by the addition of 10% trichloroactetic acid (TCA) (v/v). After washing steps to remove excessive dye, pellets were resuspended in UTC9231 (urea-thiourea CHAPS containing buffer (UTC following numbers are related to individual component molarity or detergent percentage) (9 M urea, 2 M thiourea, 3% (w/v) CHAPS, 1% (w/v) ASB14, 20 mM Tris, pH 9.5) under stirring (1600 rpm) at 30 °C in the dark for two hours. 1 µL of 0.1 mM Cye2™-NHS dye (FP- LV2330, Interchim) was added to the samples after pH adjustment to 8.6, and incubated on ice during 30 min. After stopping the reaction by adding 1 µL of 10 mM lysine, samples were subjected to 1D-SDS PAGE (sodium dodecyl sulfate polyacrylamide gel electrophoresis) (Mini-PROTEAN TGX pre-cast gels (4–15% gradient, 15wells (Biorad)), at 100 V during 15 min and 150 V until loss of the migration front. Gels were then scanned in fluorescence on a TYPHOON FLA 9500 imager (GE Healthcare). Images were analyzed according to the manufacturer’s instructions with Image Quant Total Lab (IQTL, GE Healthcare) software. Quantification was performed by on gel spotting of Cy5™-Hz dye in order to get a calibration curve.

### Measurement of proteasome activity

#### Protein extraction

After irradiation, 3000 *C*. *elegans* were suspended in 150 µL of proteasome activity lysis buffer (20 mM Tris-HCl, 5 mM MgCl_2_, 10% glycerol, 1 mM DTT, 1 mM ATP, 0.2% IGEPAL CA-630, 1 mg.ml^−1^ aprotinin, 1 mM phenylmethylsulfonylfluoride, pH 8) and homogenized during 4 min with 2000 rot.min^−1^ using Potter-Elvehjem (Eurostar digital ika labortechnik) at 4 °C. Lysates were then centrifuged (13500 g) for 15 min at 4 °C. Protein concentration was determined by method of Bradford using BSA as a standard.

#### In gel proteasome activity measurement

Native gel electrophoresis of proteasome complexes was performed with a method adapted from Myeku *et al*.^70^. 40 µg of proteins were subjected to native PAGE electrophoresis at 4 °C at 100 V during 2 h and at 150 V during 2h30 on a 3.5–8% gradient gel. Chymotrypsin-like activity was assayed following gel incubation in 50 mM Tris-HCl, pH 7.4, 5 mM MgCl_2_, 1 mM ATP, and 100 µM proteasome substrate (Succinyl-leucine-leucine-valine-tyrosine- amino-methyl-coumarin (Suc-LLVY-AMC) Sigma Aldrich) for 30 min at 37 °C. Proteasome bands were visualized under UV (Fusion FX Vilber). Activity quantification of each detected proteasome complex relative to control was done by image analysis with Image Quant Total Lab (IQTL, GE Healthcare) software. Each native gel experiment was repeated at least twice. Internal standard was used for each native gel in order to normalize the signal between the different gels.

### Statistical analysis

All statistical analyses were performed using R software version 3.2.4 and RStudio environment version 0.99.893^71^.

For reproduction endpoints, results are expressed as a mean of 20 individual biological replicates. Modeling of the data was done using a 3 parameters log-logistic model in order to determine the DE50 relative to two effect criteria *i*.*e*. the total spawning per individual and the hatching success (Dose inducing 50% of Effect compared to controls)^72^. For in gel proteasome activity and carbonylated proteins levels, results are expressed as a mean of three biological replicates (n = 3 pool of 3000 or 1000 nematodes, respectively) with corresponding standard deviation (SD) otherwise indicated. Normality (Shapiro-Wilk test) and homogeneity of data variance (Levene’s test) were tested before all statistical analysis. When these two assumptions were not found, a logarithmic transformation was applied. The effects of the considered treatments were assessed using a one-way ANOVA followed by a post-hoc pairwise comparison (Dunnet-test and Tukey-test). The significance level (alpha risk) was fixed to 0.05.

### Data availability

Data are available on request.

## Electronic supplementary material

Supplementary file
