# Supplementary material for: Precoce and opposite response of proteasome activity after acute or chronic exposure of C. elegans to γ-radiation
Source: Sci Rep. 2018 Jul 27;8:11349. doi: 10.1038/s41598-018-29033-1 (PMC6063909; doi:10.1038/s41598-018-29033-1)
Supplement: Supplementary file 1 — Supplementary file [file 41598_2018_29033_MOESM1_ESM.pdf]

Supplementary file

Precoce and opposite response of proteasome activity after acute or chronic exposure of *C. elegans* to γ-radiation

Cécile Dubois[1], Catherine Lecomte[1], Sébastien Pyr dit Ruys[1], Mira Kuzmic[1], Claire Della-Vedova[2], Nicolas Dubourg[1], Simon Galas[3], Sandrine Frelon[1*].

[1]IRSN/PSE-ENV/SRTE - Laboratoire d'ecotoxicologie des radionucléides - BP3 - 13115 St Paul lez Durance Cedex – France. [2]IRSN/PSE-ENV/SRTE – LRTA - BP3 - 13115 St Paul lez Durance Cedex – France. [3]IBMM, University of Montpellier, CNRS, ENSCM, Montpellier, France.

[*]Corresponding author: Frelon S.

tel: (+33).04.42.19.94.71 / **fax:** (+33).04.42.19.91.51 / **email:** sandrine.frelon@irsn.fr

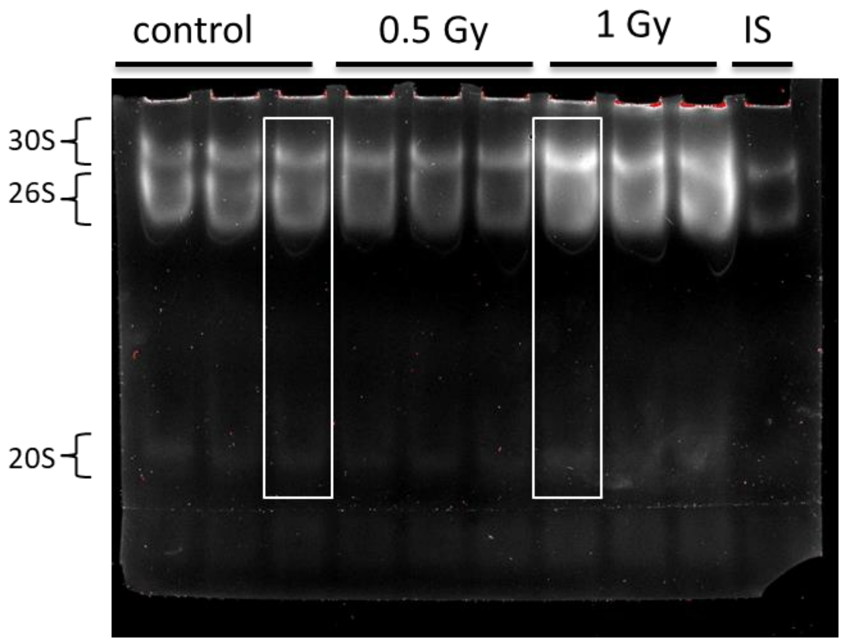

Figure S1: Full-lenght native gel electrophoresis of chronic exposure *C. elegans* protein extracts followed by in gel proteasome activity assay of the 30S 26S and 20S proteasomes. White delimitations represent cropped zone presented in Fig. 1C. IS means "internal standard". 3.5%-8% gradient gel was used. No saturation of the bands of interest was observed during revelation.

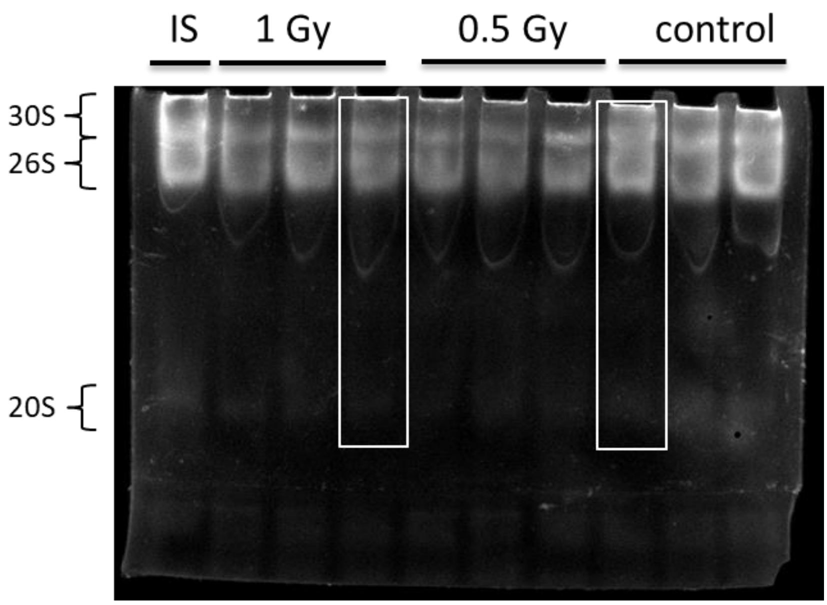

Figure S2: Full-lenght native gel electrophoresis of acute exposure *C. elegans* protein extracts followed by in gel proteasome activity assay of the 30S 26S and 20S proteasomes. White delimitations represent cropped zone presented in Fig. 3C. IS means "internal standard". 3.5%-8% gradient gel was used. No saturation of the bands of interest was observed during revelation.
